# Supplementary material for: Unobtrusive monitoring: Statistical dissemination latency estimation in Bitcoin’s peer-to-peer network
Source: PLoS One. 2020 Dec 10;15(12):e0243475. doi: 10.1371/journal.pone.0243475 (PMC7728217; doi:10.1371/journal.pone.0243475)
Supplement: Attachment — Submitted filename: response to reviewers.pdf [file pone.0243475.s001.pdf]

Dear Editor,

we thank the reviewers and editors for their generous comments to improve the manuscript. We have edited the manuscript to address their concerns.

In particular we addressed the points raised in the following way:

1) Title changes: We improved the title to make it more specific and provide a better insight into the contents of the manuscript.
2) Abstract improvements: We amended the abstract to give further context for readers.
3) Acronyms: We incorporated the recommended introduction of acronyms on first use.
4) Numbering of equations: We numbered the equations in the manuscript.
5) Conclusion Improvements: We added further context and more precise statements in the conclusion, as recommended by the reviewer.
6) Reference improvements: We added missing information for journals. Unfortunately, not all relevant publications regarding blockchains are academically published. We cleaned up the citations and included information presented in the PLOS ONE reference guide to easily find the referenced works.
7) Overuse of “we”: We reduced the usage of “we” sentences throughout the manuscript as recommended by the reviewer.
8) Additional writing: We corrected some grammar issues and typos.
9) Unlabeled Y Axis: We added labels to the figures missing Y Axis labels. We removed one distribution from one of the graphs, due to errors with the underlying software library. The units of axis are mostly numeric, as common in similar publications in PLOS ONE.
10) Additional images: Reviewed and updated image quality to 300dpi using PACE.

We believe this addresses the issues raised by the reviewers.

Best regards,
David Mödinger
